# Sex-Specific Differences in the Physiological and Biochemical Performance of Arbuscular Mycorrhizal Fungi-Inoculated Mulberry Clones Under Salinity Stress

**DOI:** 10.3389/fpls.2021.614162

**Published:** 2021-03-18

**Authors:** Yan-Hong Wang, Nai-Li Zhang, Min-Qiang Wang, Xiao-Bin He, Zhi-Qiang Lv, Jia Wei, Xiu Su, Ai-Ping Wu, Yan Li

**Affiliations:** ^1^State Key Laboratory of Subtropical Silviculture, Zhejiang A&F University, Hangzhou, China; ^2^College of Forestry, Beijing Forestry University, Beijing, China; ^3^Institute of Sericulture and Tea, Zhejiang Academy of Agricultural Sciences, Hangzhou, China; ^4^Ecology Department, College of Resources and Environment, Hunan Provincial Key Laboratory of Rural Ecosystem Health in Dongting Lake Area, Hunan Agricultural University, Changsha, China

**Keywords:** arbuscular mycorrhizal fungi, *Funneliformis mosseae*, *Morus alba*, salinity stress, sex-specific differences, dioecy

## Abstract

Arbuscular mycorrhizal fungi (AMF) are often considered bioameliorators. AMF can promote plant growth under various stressful conditions; however, differences between male and female clones in mycorrhizal strategies that protect plants from the detrimental effects of salinity are not well studied. In this study, we aimed to examine the interactive effects of salinity and AMF on the growth, photosynthetic traits, nutrient uptake, and biochemical responses of *Morus alba* males and females. In a factorial setup, male and female *M. alba* clones were subjected to three salinity regimes (0, 50, and 200 mM NaCl) and planted in soil with or without *Funneliformis mosseae* inoculation. The results showed that NaCl alone conferred negative effects on the growth, salinity tolerance, photosynthetic performance, and shoot and root ionic ratios (K^+^/Na^+^, Ca^2+^/Na^+^, and Mg^2+^/Na^+^) in both sexes; in contrast, mycorrhizal inoculation mitigated the detrimental effects of salinity. Furthermore, the mycorrhizal effects were closely correlated with Mn^2+^, proline, and N concentrations. Females benefited more from AMF inoculation as shown by the enhancements in their biomass accumulation, and N, proline, K^+^, Mg^2+^, Fe^2+^, Zn^2+^, and Mn^2+^ concentrations than males with mycorrhizal inoculation under saline conditions. In comparison, male plants inoculated with AMF showed improvements in biomass allocated to the roots, P, and peroxidase concentrations under saline conditions. These sex-specific differences suggest that male and female mulberry clones adopted different mycorrhizal strategies when growing under saline conditions. Overall, our results provide insight into the sex-specific difference in the performance of AMF-associated mulberry clones, suggesting that female mulberry could be more suitable for vegetation remediation than the male one, due to its higher salinity tolerance.

## Introduction

Soil salinity, a major concern in agriculture and forestry (Wu et al., 2016; van Zelm et al., 2020), is spreading and continuously worsening with the ongoing global warming (Rengasamy, 2006; Munns and Tester, 2008; Ondrasek and Rengel, 2021). In China, nearly 10% (1 × 10^8^ ha) of arable land is saline (Wu et al., 2012). When exposed to salinity, plant growth is stunted with biomass production loss, nutrient imbalance, and oxidative damage (Mahajan and Tuteja, 2005; Zhao et al., 2020). However, most relevant studies have focused on herbaceous plants (72.4%), and less than 19.5% of the studies were relevant to trees (Chandrasekaran et al., 2014). Given that herbaceous and tree species have different physiological and biochemical characteristics, our ability of comprehensively understanding saline stress on tree species is limited (Giri et al., 2007; Kapoor et al., 2019).

In terrestrial ecosystems, approximately 6% (14,620 of 240,000) of angiosperm species are dioecious (Renner and Ricklefs, 1995; Juvany and Munné-Bosch, 2015), most of which are wind-pollinated woody species (Chen et al., 2010). Renner and Ricklefs (1995) suggested that the differences in the reproductive strategies of dioecious plants could induce different resource demands within sexes, which would lead to sex-specific specializations of growth and eco-physiological traits. Over the past century, compelling evidence has confirmed secondary sexual dimorphism in physiological and biochemical traits under various stresses (Juvany and Munné-Bosch, 2015). It is worth noting that male and female individuals of plant species have been documented to respond differently to salinity stress (Juvany and Munné-Bosch, 2015; Melnikova et al., 2017; Hao et al., 2020). However, these sex-specific differences in physiological traits are not well understood and change widely within species (Nicotra et al., 2003; Chen et al., 2010; Li et al., 2013; McKown et al., 2017). It is generally accepted that male plants can perform better than females under unfavorable conditions (Juvany and Munné-Bosch, 2015; Melnikova et al., 2017). Nonetheless, in some cases, female plants exhibit augmented growth under adverse conditions compared with males (McKown et al., 2017). Owing to this discrepancy, exploring the mechanism underlying the sex-specific differences exhibited under saline conditions is necessary.

Arbuscular mycorrhizal (AM) fungi (AMF) can form symbiotic unions with 80% of terrestrial plants (Smith and Read, 2008) and occur naturally in saline soils (Juniper and Abbott, 1993; Chandrasekaran et al., 2014). AMF can increase the salt tolerance of host plants under various salt concentrations (Hoeksema et al., 2010; Chandrasekaran et al., 2014; Kapoor et al., 2019; Ingraffia et al., 2020). Mycorrhizal fungi abrogate the detrimental effects of salinity through several possible mechanisms, such as improving photosynthetic efficiency and nutrient acquisition, preserving the ionic homeostasis and osmotic equilibrium in plants, and enhancing the antioxidant system to prevent damage from reactive oxygen species (Ruiz-Lozano et al., 1996; Evelin et al., 2009; Kapoor et al., 2019). Moreover, AMF have been reported to confer different effects on male and female dioecious plants under various saline conditions (Varga and Kytöviita, 2008; Reuss-Schmidt et al., 2015; Wu et al., 2016; Melnikova et al., 2017). These sex-specific differences in mycorrhizal effects may be induced by differences in plant species and their mycorrhizal dependencies (Melnikova et al., 2017).

*Morus alba* L. of the Moraceae family is an economically important tree species used for multiple purposes throughout China. It is well known in sericulture because its leaves are high in protein content and widely used for raising silkworms (Kashyap and Sharma, 2006; Fan et al., 2014; Zhang et al., 2020). Currently, with the transfer of the mulberry industry from southeastern China, the developed area, to inland China, where the soil is arid and salinized (Ke et al., 2009), salt tolerance would be a highly desired plant characteristic. It has been found that *M. alba* cannot grow well in saline conditions (Kumar et al., 2003; Sun et al., 2009). Furthermore, the highly heterozygous and dioecious nature of this genus makes the development of salt-tolerant cultivars using conventional techniques difficult (Kashyap and Sharma, 2006). Given that *M. alba* is a typical mycorrhizal plant and the mycorrhizal colonization of its roots is up to 79% in the field (Shu et al., 2011), we hypothesized that inoculating plants of this species with AMF would help alleviate the detrimental effects of salinity. Moreover, it has been reported that there exist sex-specific differences in the physiological traits of *M. alba*, with females being more adaptive to unfavorable conditions than males (Zheng et al., 2018; Lin et al., 2019). We also hypothesized that male and female mulberry plants have evolved different mycorrhizal strategies when growing under saline stress, and we expected the females to benefit more from mycorrhizas than males in terms of nutrient uptake. To examine the impact of AMF and salt on male and female *M. alba* clones, we conducted a pot experiment to determine their growth, photosynthetic trait, nutrient uptake, and biochemical responses. This research would help to unravel the underlying adaptive mechanisms in two sexes of *M. alba* growing in saline habitats and gain further insight into the secondary sexual dimorphism under stressful conditions.

## Materials and Methods

### Plant Material and Experimental Design

NaCl is the most soluble and widespread salt compound, and soils with more than 40 mM NaCl are classified as saline (Munns and Tester, 2008). We designed a full factorial experiment that included three NaCl regimes (0, 50, and 200 mM), two mycorrhizal inoculation types (NM, non-inoculated with mycorrhizal fungus; AM, inoculated with *Funneliformis mosseae*), and two sexes (male and female cuttings of *M. alba*). This design resulted in 12 treatment combinations that were arranged in a randomized complete block design with six replications. On March 16, 2017, 160 healthy *M. alba* cuttings (80 females and 80 males) were collected from the germplasm resources nursery at the Institute of Sericulture and Tea of Zhejiang Academy of Agricultural Sciences in Hangzhou, China. These cuttings originated from 160 F1 individuals (80 females and 80 males), which resulted from the artificial cross-pollination of *M. alba* cv. “tongxiangqing” and *M. alba* cv. “dazhongsang” in 2007. Then, these cuttings were moved into a greenhouse, sterilized in 5% sodium hypochlorite solution for 15 min, and cultured in trays with background soil (3:1 mixture of coarse sand and peat), which was sterilized twice by autoclaving for 2 h, with a 1-day rest period in between (Wang et al., 2018). The greenhouse was located nearby Zhejiang A&F University in southeastern China (30°14′ N, 119°42′ E). Forty-nine days later, 36 male and 36 female clones (30 cm in height, 20 cm in root length, and with three leaves per plant) were randomly chosen to be used for this experiment.

All male and female clones, which were 49 days old, were planted separately in plastic pots (21 cm × 22 cm × 17 cm) filled with 4 kg of soil medium subjected to gamma irradiation in a dose of 25 kGy on May 5, 2017 (McNamara et al., 2003). The potting medium used was a mixture of local field soil and peat in a volume ratio of 3:1. The local soil belongs to the yellow-red soil class, which is a kind of Hapludult soil in soil taxonomy (Gong, 2001). The soil mixture had the following properties: bulk density, 1.6 g cm^–3^; organic matter, 22.31 mg g^–1^; total N, 1.189 mg g^–1^; total P, 0.488 mg g^–1^; electrical conductivity (EC), 0.21 dS m^–1^; and pH, 4.8 (soil/water = 1:5). The *F. mosseae* inoculum (BGC HUN03B) was obtained from the Bank of Glomeromycota in China of the Institute of Plant Nutrients and Resources of Beijing Municipal Academy of Agriculture and Forestry Science. *F. mosseae* was initially isolated from the rhizosphere of *Roegneria kamoji* Ohwi in Chenzhou, Hunan Province, China, and multiplied for 5 months using *Sorghum bicolor* L. as a trap plant (Wang et al., 2018). Before transplanting the clones into the pots, 40 g of inoculum (containing ∼70 spores per 10 g of soil) was added to the medium in each pot at a 10-cm depth. For the controls, 40 g of sterilized inoculum (121°C, 2 h in the autoclave) was added to the pots. Finally, all pots received 40 ml of inoculated soil suspension that was sieved through a 25-μm filter to reintroduce the native microbial populations (excluding AMF) (Evelin et al., 2012).

When all the *M. alba* clones were established after 1 month, NaCl treatment was started on June 6, 2017. To prevent the effects of osmotic shock on the development of plant fine roots and AMF establishment, 195 ml of the prescribed NaCl solution was administered gradually to each pot for 7 days in the experiment following the protocol of Evelin et al. (2012). This resulted in EC 0.21 (control without salt stress), 2.6, and 9.6 dS m^–1^ for the 0, 50, and 200 mM NaCl treatments, respectively. The plants were watered with distilled water, and leaching was prevented. To preserve the salinity treatments near the target levels, the soil EC in each pot was monitored using a conductivity meter (FE38; Mettler-Toledo, Greifensee, Switzerland) and adjusted once per 2 weeks. To ensure the development of plants, 10 ml of adjusted Hoagland solution was added to all pots weekly (Wang et al., 2018). During the experiment, the mean temperature and relative humidity in the greenhouse were 33.2°C and 73.7%, respectively (Thermo Datalogger; Campbell Scientific, Logan, UT, United States). On October 16, 2017, the plants were harvested 132 days after salinity stress when they were in the vegetative phase in all the treatments, and their dry weights were obtained after oven-drying at 70°C for 48 h.

### Determination of Leaf Photosynthesis

From October 12 to October 14, 2017, the leaf net photosynthetic rate (*A*), stomatal conductance (*g*_*s*_), and transpiration rate (*E*) were measured between 8:00 and 11:30 am, when the peak of maximum photosynthetic rates was found at each survey (León-Sánchez et al., 2016), using the LI-6400XT photosynthesis system (Li-Cor Biosciences, Lincoln, NE, United States) (Zhang et al., 2018). The leaf photosynthetic measurements were conducted on the fully sun-exposed leaves of three plants randomly selected from each treatment combination. During these measurements, air CO_2_ concentration, photosynthetically active radiation, leaf temperature, and relative humidity were set at 400 μmol mol^–1^, 1,200 μmol m^–2^ s^–1^, 25°C, and 70%, respectively. Additionally, instantaneous water use efficiency (WUE) was determined as *A*/*E*.

### Determination of Proline, Soluble Protein, and Enzyme Activities of Leaf

After harvesting, the proline, soluble protein (SP), and peroxidase (POD) concentrations of the leaves of three plants that were randomly selected from each treatment were immediately determined using spectrophotometric method with commercial kits (Nanjing Jiancheng Institute of Bioengineering, Nanjing, China) (Guo et al., 2020).

### Determination of Elemental Composition

Dried shoot and root samples of the three plants that were randomly selected from each treatment were ground separately, and their macro- and micronutrient concentrations were analyzed. Nitrogen and P concentrations were determined using the Kjeldahl method and ammonium molybdate blue method, respectively (Allen, 1989), whereas K^+^, Na^+^, Ca^2+^, Mg^2+^, Fe^2+^, Zn^2+^, and Mn^2+^ concentrations were measured following the methods described by Colla et al. (2008) using an atomic absorption spectrophotometer (AA7000; Shimadzu, Tokyo, Japan).

### Determination of Mycorrhizal Colonization

Root samples (0.5 g) obtained from each plant in each treatment combination were cleared with 10% KOH at 90°C for 1.5 h, acidified in 1 M HCl for 5 min, and stained with 0.05% trypan blue (Wang et al., 2018). Then, 200 stained root segments were microscopically examined to analyze the mycorrhizal root colonization using the gridline intercept method (Giovannetti and Mosse, 1980).

### Data Analysis

To quantify the mycorrhizal effects, the mycorrhizal growth response (MGR) was calculated according to a previously reported method (Zhang et al., 2016):

$$MGR\left(\%\right)=\frac{D\,W_{A\,M\,F}-\bar{D\,W_{n\,o\,n-A\,M\,F}}}{\bar{D\,W_{n\,o\,n-A\,M\,F}}}\times 100$$

where *DW*_*AMF*_ is the total dry weight of a plant inoculated with AMF and $\bar{D\,W_{n\,o\,n-A\,M\,F}}$ is the mean dry weight of plants that were not inoculated with AMF. A positive MGR value represents growth promotion in the host plant that was inoculated with AMF, whereas a negative MGR value represents an inhibitory effect of AMF on the host.

Additionally, the salinity tolerance (ST) of each plant was computed as follows (Zhang et al., 2016):

$$ST\left(\%\right)=\frac{D\,W_{s\,s}-\bar{D\,W_{c\,k}}}{\bar{D\,W_{c\,k}}}\times 100$$

Where *DW*_*ss*_ is the dry weight of salinity-stressed plants and $\bar{D\,W_{c\,k}}$ is the mean dry weight of the controls. In the study, a higher ST value would represent a higher tolerance capacity.

To estimate the responses of plant parameters to sex, salt, AMF, and their combinations, we performed a three-way ANOVA in IBM SPSS Statistics for Windows, version 23.0 (IBM Corp., Armonk, NY, United States). Meanwhile, two-way ANOVA was performed to detect the effects of sex, salt, and their combinations on root colonization and MGR. Before analysis, all the root/shoot ratio, POD, Na^+^, and Fe^2+^ concentrations of whole plant, and shoot and root nutrient ratio (K^+^/Na^+^, Ca^2+^/Na^+^, Mg^2+^/Na^+^) data were log10-transformed. The *g*_*s*_ and Fe^2+^ data in the whole plant were square root-transformed to conform to the requirements of Levene’s test for equal variances and the Shapiro–Wilk test for normality; the rest of the data were analyzed without transformation. Differences between individual means were compared using the least significant difference test at *p* < 0.05. Principal component analysis was performed using the package “vegan” in R 4.0.2 (R Core Team, 2020) to determine the relationship strength of the investigated parameters.

## Results

### Mycorrhizal Colonization and Plant Growth

**FIGURE 1:**
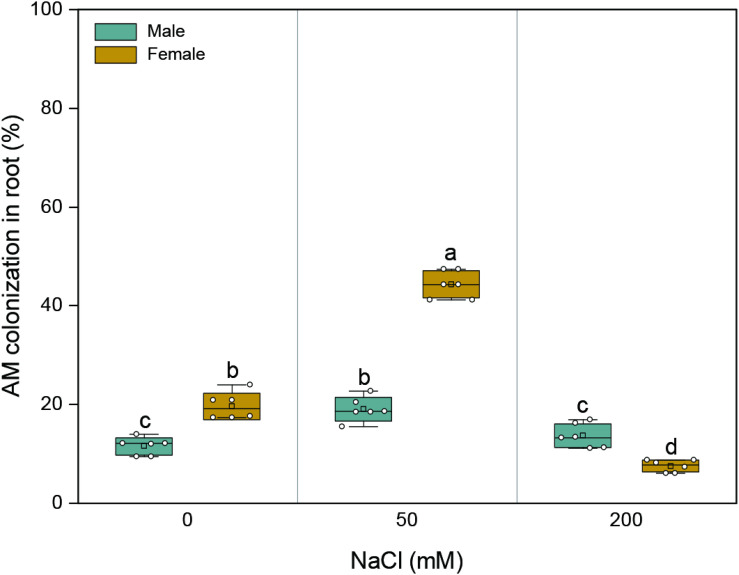
Arbuscular mycorrhizal colonization of roots in about 7-month-old males and females of *Morus alba* inoculated with *Funneliformis mosseae* after 132 days of salinity treatment. Values are presented as means ± SD (*n* = 6). Two-way ANOVA is performed to compare the effects of sex and salt and their interactions in both males and females. Different letters indicate a significant difference according to least significant difference at *p* < 0.05.

AMF colonization was not detected in the roots of *M. alba* clones grown in soils without the AMF inoculum. With an increase in salinity, AMF colonization in the roots of both sexes initially increased and subsequently decreased (Figure 1 and Supplementary Figure 1). The mycorrhizal colonization of female plants varied from 7.5 to 44.3% throughout the duration of salinity treatments, while that of male plants varied from 11.6 to 19.1%. At 0 and 50 mM NaCl, the mycorrhizal colonization of female plants was higher than that of male plants by 70 and 132%, respectively; at the highest saline condition, the mycorrhizal colonization of females was lower than those of males by 45% (Figure 1). Significant effects of sex (*F*_1,30_ = 143.395, *p* = 0.000), salt (*F*_2,30_ = 282.465, *p* = 0.000), and their combination (*F*_2,30_ = 143.427, *p* = 0.000) on root colonization were observed.

**FIGURE 2:**
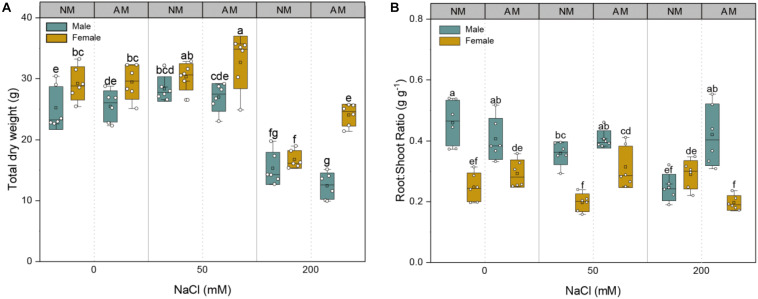
Effects of arbuscular mycorrhizal fungus on total dry weight **(A)** and root/shoot ratio **(B)** in males and females of *Morus alba* under saline conditions. NM and AM represent inoculation with no mycorrhizal fungi and with *Funneliformis mosseae*, respectively. Bars represent means ± SD (*n* = 6). Three-way ANOVA is performed to compare the effects of sex, salt, and AM inoculation and their interactions in both males and females. Different letters indicate a significant difference according to least significant difference at *p* < 0.05.

The total dry weights of both sexes were severely affected by salinity (*p* = 0.000) (Supplementary Table 1). At the highest NaCl concentration (200 mM NaCl), the total dry weights of male and female plants with non-mycorrhizal inoculation (NM plants) decreased substantially by 39.3 and 42.7%, respectively, whereas those of male and female plants inoculated with AMF (AM plants) decreased significantly by 51.4 and 18.4%, respectively, in comparison with the corresponding non-saline controls (Figure 2A). Under non-saline conditions, mycorrhizal inoculation had no effects on the root/shoot ratios of either sex. At 50 mM NaCl, mycorrhizal inoculation had no effects on the root/shoot ratio of male plants but significantly increased that of female plants by 60.2%. At 200 mM NaCl, fungal colonization significantly increased the root/shoot ratio of male plants by 70.3% but decreased that of female plants by 32.6% compared with that of NM plants (Figure 2B). These findings indicate that mycorrhizal inoculation could effectively alleviate the detrimental effects of salinity on the biomass accumulation of female plants but that the mycorrhizal efficiency on root/shoot ratio varied with saline conditions. Significant interactive effects of sex, salt, and AMF on the total dry weight (*p* = 0.005) and root/shoot ratio (*p* = 0.000) were observed (Supplementary Table 1).

The MGR of males decreased significantly as salinity increased, whereas that of females increased greatly (Supplementary Figure 2). At lower NaCl conditions, there were no sex-specific MGR differences. In contrast, at the highest NaCl concentration, the MGR of female plants inoculated with AMF was markedly higher than that of males (Supplementary Figure 2A). Increasing salinity induced a considerable decline in the ST of both sexes (Supplementary Figure 2B). At 200 mM NaCl, the ST values of males inoculated with AMF decreased by 30.6%, but that of females inoculated with AMF increased by 56.9% compared with that of NM plants. The responses in MGR and ST suggest that female plants can benefit more from mycorrhizal inoculation and behave better in salt tolerance by building associations with AMF under saline conditions. A significant interactive effect of sex and salt on MGR (*p* = 0.000) and a significant interactive effect of sex, salt, and AMF on ST (*p* = 0.037) were noted (Supplementary Table 1).

### Leaf Photosynthesis

**FIGURE 3:**
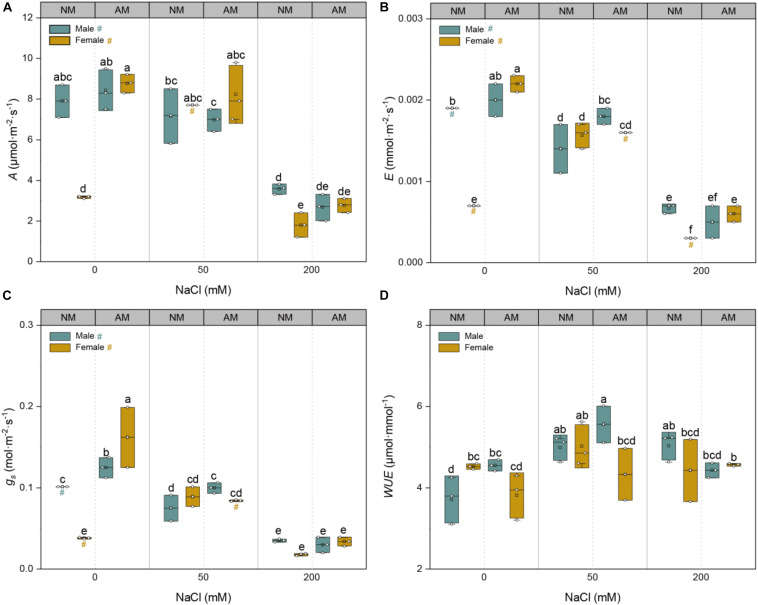
Effects of arbuscular mycorrhizal fungus on net photosynthetic rate (*A*) **(A)**, stomatal conductance (*g*_*s*_) **(B)**, transpiration rate (*E*) **(C)**, and instantaneous water use efficiency (WUE) **(D)** in males and females of *Morus alba* under saline conditions. NM and AM represent inoculation with no mycorrhizal fungi and with *Funneliformis mosseae*, respectively. Values are presented as means ± SD (*n* = 3). Three-way ANOVA is performed to compare the effects of sex, salt, and AM inoculation and their interactions in both males and females. Different letters indicate a significant difference according to least significant difference at *p* < 0.05.

Increased salinity levels induced a gradual decline in the leaf net photosynthetic rate (*A*), transpiration rate (*E*), and stomatal conductance (*g*_*s*_) values of both sexes irrespective of female NM plants (Figures 3A–C). Under non-saline conditions, mycorrhizal inoculation significantly increased *A*, *E*, and *g*_*s*_ of females but only *g*_*s*_ of males. Under 50 mM NaCl treatment, AMF inoculation had positive effects on the *E* and *g*_*s*_ of males but not on those of females. Under 200 mM NaCl treatment, AMF inoculation significantly increased the *E* of females by approximately twofold but had no effects on males. Mycorrhizal inoculation only had significant effects on the WUE of males under non-saline conditions (Figure 3D). Notably, mycorrhizal inoculation conferred more effects on the photosynthetic ability of females than males. A significant interactive effect of sex, salt, and AMF on *A* (*p* = 0.006), *E* (*p* = 0.000), *g*_*s*_ (*p* = 0.000), and WUE (*p* = 0.009) was detected (Supplementary Table 1).

### Proline, SP, and Antioxidant Capacity

**FIGURE 4:**
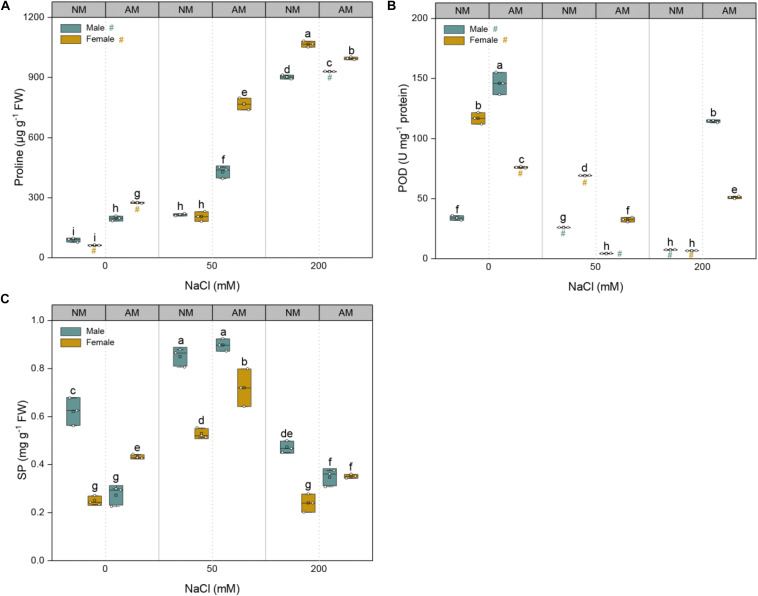
Effects of arbuscular mycorrhizal fungus on proline concentrations **(A)**, peroxidase **(B)**, and soluble protein (SP) **(C)** in males and females of *Morus alba* under saline conditions. NM and AM represent inoculation with no mycorrhizal fungi and with *Funneliformis mosseae*, respectively. Values are presented as means ± SD (*n* = 3). Three-way ANOVA is performed to compare the effects of sex, salt, and AM inoculation and their interactions in both males and females. Different letters indicate a significant difference according to least significant difference at *p* < 0.05.

The exposure of plants to NaCl resulted in significant increases in the proline concentrations of both sexes; the POD and SP concentrations varied with salinity levels (Figure 4). The proline concentrations of AM plants of both sexes were higher than those of NM plants at lower saline conditions (0 and 50 mM NaCl). Moreover, the proline concentrations of females inoculated with AMF were higher by 40.1, 79.3, and 7.1% than those of males inoculated with AMF at 0, 50, and 200 mM NaCl, respectively (Figure 4A). With an increase in salinity, the POD concentrations of NM plants of both sexes decreased significantly, whereas that of AM plants initially decreased and subsequently increased in both sexes (Figure 4B). At a non-saline condition and 200 mM NaCl, the POD concentrations of males inoculated with AMF were higher than those of females inoculated with AMF; however, at 50 mM NaCl, those of females inoculated with AMF were higher than those of males with mycorrhizal inoculation. The SP concentrations of both sexes initially increased and subsequently decreased as salinity increased (Figure 4C). At all salinity levels, the SP of females inoculated with AMF was consistently higher than that of females grown without AMF, whereas this kind of positive mycorrhizal efficacy was not detected in males. These findings indicate that mycorrhizal association alleviate the salinity-caused osmotic stress in females by regulating the osmotic potential with accumulation of more proline and SP at all salinity levels, whereas the mycorrhizal benefits for males were mainly embodied in an increase of POD at the highest saline condition. Significant interactive effects of sex, salt, and AMF on proline (*p* = 0.000), POD (*p* = 0.000), and SP (*p* = 0.000) were observed (Supplementary Table 1).

### Mineral Nutrient Concentrations

**FIGURE 5:**
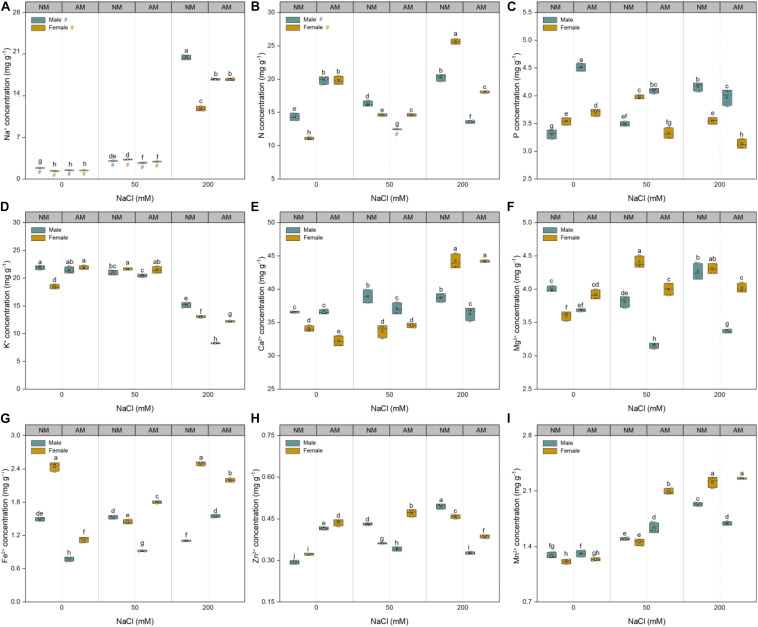
Effects of arbuscular mycorrhizal fungus on Na^+^ **(A)**, N **(B)**, P **(C)**, K^+^ **(D)**, Ca^2+^ **(E)**, Mg^2+^ **(F)**, Fe^2+^ **(G)**, Zn^2+^ **(H)**, and Mn^2+^ **(I)** of whole plant in males and females of *Morus alba* under saline conditions. NM and AM represent inoculation with no mycorrhizal fungi and with *Funneliformis mosseae*, respectively. Bars represent means ± SD (*n* = 3). Three-way ANOVA is performed to compare the effects of sex, salt, and AM inoculation and their interactions in both males and females. Different letters indicate a significant difference according to least significant difference at *p* < 0.05.

The sodium concentrations of plants of both sexes showed a linear increase with increasing soil salinity (Figure 5A). At saline conditions, the Na^+^ concentrations of males inoculated with AMF were lower than those of males grown without AMF. At 50 mM NaCl, the Na^+^ concentrations of females inoculated with AMF were lower than those of females grown without AMF but higher than those of their counterparts at 200 mM NaCl. Compared with that of controls, the nutrient uptake of the treated plants showed variable changes with increasing salinity (Figure 5). Under 50 mM NaCl treatment, mycorrhizal inoculation significantly increased the N, Fe^2+^, Zn^2+^, and Mn^2+^concentrations of female plants and the P and Mn^2+^concentrations of male plants but decreased the N, Ca^2+^, Fe^2+^, Zn^2+^, and Mg^2+^ concentrations of male plants and P and Mg^2+^ concentrations of female plants. Under 200 mM NaCl treatment, fungal colonization significantly increased the Fe^2+^ concentrations of males, whereas it decreased the N, P, K^+^, Mg^2+^, Fe^2+^, and Zn^2+^ concentrations of females and N, P, K^+^, Ca^2+^, Mg^2+^, Zn^2+^, and Mn^2+^ concentrations of males. Furthermore, under saline conditions, the N, K^+^, Mg^2+^, Fe^2+^, Zn^2+^, and Mn^2+^ concentrations of females inoculated with AMF were higher than those of males inoculated with AMF, whereas the P concentrations of males inoculated with AMF were higher than those of females inoculated with AMF. Collectively, these results showed that AMF conferred more beneficial effects on the nutrient uptake of females than males under saline conditions.

**FIGURE 6:**
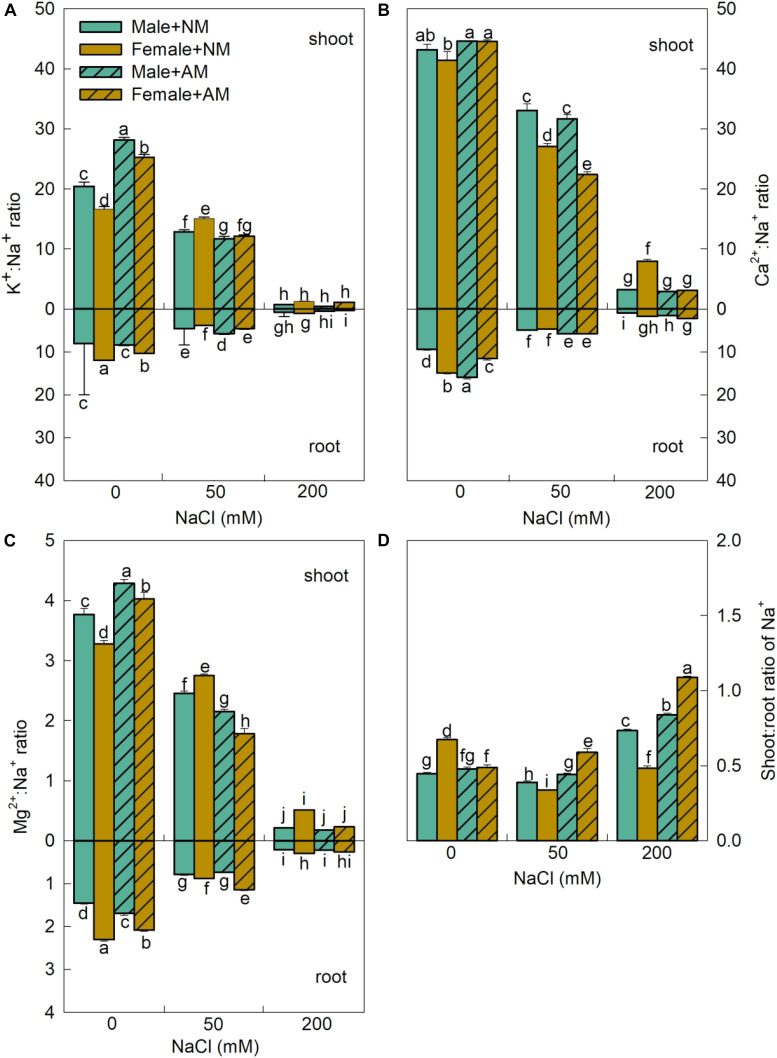
Effects of arbuscular mycorrhizal fungus on K^+^/Na^+^ ratio **(A)**, Ca^2+^/Na^+^ ratio **(B)**, Mg^2+^/Na^+^ ratio **(C)** of shoots and roots, and shoot/root ratio of Na^+^ **(D)** in males and females of *Morus alba* under saline conditions. NM and AM represent inoculation with no mycorrhizal fungi and with *Funneliformis mosseae*, respectively. Values are presented as means ± SD (*n* = 3). Three-way ANOVA is performed to compare the effects of sex, salt, and AM inoculation and their interactions in both males and females. Different letters indicate a significant difference according to least significant difference at *p* < 0.05.

Meanwhile, the K^+^/Na^+^, Ca^2+^/Na^+^, and Mg^2+^/Na^+^ ratios of shoot and root in both sexes decreased significantly with increasing salinity, and these ratios were significantly higher in shoots than in roots (Figures 6A–C), indicating that the root is more sensitive to salt stress than the shoot. The mycorrhizal efficacies on ionic ratios varied with saline conditions and plant organs. The root K^+^/Na^+^ ratios of males inoculated with AMF were higher than those of females inoculated with AMF at 50 mM NaCl. However, the root Mg^2+^/Na^+^ ratios of females inoculated with AMF at 50 mM NaCl and the root Ca^2+^/Na^+^ ratios of females inoculated with AMF at 200 mM NaCl were higher than those of males. The shoot/root Na^+^ ratios varied with sexes and salinity levels (Figure 6D). Under saline conditions, the shoot/root Na^+^ ratios of females inoculated with AMF were consistently higher than those of males inoculated with AMF. These results suggest that there exist sex-specific responses in mycorrhizal strategies on nutrient ratios, and more Na^+^ was transferred to the shoots, especially in females inoculated with AMF. Significant interactive effects of sex, salt, and AMF on the nutrient concentrations and ionic ratios were found (Supplementary Table 2).

### Principal Component Analysis of Sex-Specific Differences

Principal component analysis was performed to assess the relationships among growth, photosynthetic properties (*A*, *E*, *g*_*s*_, and WUE), MGR, biochemical parameters (proline, SP, and POD), nutrient uptake, and the combinations of sex, salinity, and AMF (Supplementary Figure 3). The first two axes in the biplot explain 60.3% of the total variation. The coefficients of shoot Ca^2+^/Na^+^ and Mg^2+^/Na^+^ ratios and the *A*, *E*, and K^+^ concentrations were the highest in the first principal component and that of SP and POD was the highest in the second principal component. The individual and combined sex, salinity, and AMF treatments were separated significantly from the controls. It was obvious that the mycorrhizal effects exhibited significant positive correlations with Mn^2+^, proline, and N concentrations.

## Discussion

In this study, the exposure of plants to NaCl alone exerted negative effects on growth, ST, photosynthetic properties, nutrient acquirement, and K^+^/Na^+^, Ca^2+^/Na^+^, and Mg^2+^/Na^+^ of shoot and root ratios in both *M. alba* sexes. In contrast, mycorrhizal inoculation mitigated the detrimental effects of salinity, which supports our first hypothesis that AMF inoculation can alleviate the detrimental effects of salt stress. Furthermore, we found multifaceted sex-specific differences in plant growth traits and physiological parameters induced by AMF colonization, which supported the other hypothesis that the two *M. alba* sexes adopted different mycorrhizal strategies when growing under saline conditions. Our findings provide insights into the fact that female plants inoculated with AMF benefited more from AMF inoculation than male plants in response to salinity.

It is reported that AM symbiosis can improve salinity tolerance in host plants through a combination of physiological, biochemical, and nutritional effects (Evelin et al., 2009; Chandrasekaran et al., 2014), which is proven by our findings, but there exist sex-specific mycorrhizal strategies in alleviating salt stress. Our results showed that the total dry weight of female *M. alba* plants inoculated with *F. mosseae* was higher than that of male plants grown under saline conditions (Figure 2), which is in line with cottonwood (Nielsen et al., 2010) and *Antennaria dioica* (Varga and Kytöviita, 2012). Higher biomass accumulations in female plants mean that more carbohydrates could be directed toward the growth of the AMF symbiont and subsequently lead to the dilution of Na^+^ and Cl^–^ (Kapoor et al., 2019). Accordingly, the mycorrhizal efficiency of female plants was positive, whereas that of male plants was negative, with increasing levels of NaCl (Supplementary Figure 2A). Furthermore, at the highest saline condition, the salt tolerance of females inoculated with AMF was markedly higher than that of males inoculated with AMF (Supplementary Figure 2B), indicating that AMF colonization mainly improved the salt tolerance of females but not of males. Obviously, in this study, females benefited more from mycorrhizal inoculation than males under saline conditions, which is consistent with the results of *Distichlis spicata* (Reuss-Schmidt et al., 2015). It is essential to point out that both *M. alba* sexes may adopt different strategies to acclimate to NaCl stress conditions: more biomass accumulated in female plants, whereas more biomass was allocated to the roots of male plants. This sexual dimorphism in growth may be related to the differences in reproductive costs between males and females (Juvany and Munné-Bosch, 2015).

In order to adapt to saline environment, plants would undergo a series of physiological and biochemical changes which are beneficial to the improvement of salt tolerance (van Zelm et al., 2020; Wang et al., 2020), for example, increases in osmolyte concentrations like proline and SP and antioxidant enzymes like POD (Zhao et al., 2020). In our experiment, salt increased proline accumulation, and AM plants accumulated more proline than NM plants when exposed to salinity stress (Figure 4A), which is consistent with the results of previous studies (Chandrasekaran et al., 2014; Lu et al., 2014). However, some studies reported that mycorrhizal plants accumulate lesser proline than their NM counterparts under saline conditions (Evelin and Kapoor, 2014; Latef and He, 2014). Moreover, males and females showed different responses to salt and AMF inoculation in terms of osmolyte accumulation. Female *M. alba* plants accumulated more proline than males that received an identical AMF inoculation (Figure 4A). The SP concentrations of females inoculated with AMF was higher than that of NM ones when subjected to NaCl stress (Figure 4C). In contrast, the POD concentrations of males inoculated with AMF was greatly higher than that of females inoculated with AMF under the highest saline condition (Figure 4B), which is in agreement with the study of *Populus cathayana* conducted by Wu et al. (2016). Collectively, females and males of *M. alba* adopt different mycorrhizal strategies in improving salt tolerance, taking into account the induction of osmotic and oxidative stress by salinity. The females inoculated with AMF follow the way of accumulating more proline and SP under saline conditions, whereas males inoculated with AMF take the way of possessing more proline at all saline conditions and more POD at the severest saline condition in comparison with NM plants. These differences in osmoregulatory capacity and antioxidant ability suggest that the adaptive mechanisms induced by mycorrhizal inoculation are sex specific as well.

A marked effect of AMF on nutrient uptake was considered one of the main reasons for improvement of salt tolerance in salt-affected plants colonized by mycorrhizal fungi (Evelin et al., 2009). In the present study, the fact that the Na^+^ concentrations of males inoculated with AMF were consistently lower than those of male plants grown without AMF at all saline conditions indicates that symbiosis with AMF can inhibit Na^+^ uptake in males under saline conditions (Figure 5A). The response of Na^+^ in male plants inoculated with AMF was consistent with the findings in citrus seedlings (Wu et al., 2010) and *Zelkova serrate* seedlings (Wang et al., 2019) but was opposite to the response in female plants inoculated with AMF at the highest saline condition. The extent of plant sensitivity to salinity depends not only on Na^+^ uptake but also on its distribution in plants (Evelin et al., 2012). In this study, our results showed that, although there was a continuous increase in shoot/root Na^+^ ratio in both sexes with AMF inoculation (Figure 6), the shoot/root Na^+^ ratio increase that resulted from mycorrhizal inoculation was considerably lower in males than in females. These results demonstrate that male plants have a better ability to restrain the translocation of Na^+^ from the roots to the shoots than female plants do (Chen et al., 2010).

Nitrogen is the mineral element that plants absorb in the highest amounts, and changes in N uptake influence many important metabolic processes (Evelin et al., 2009). In this study, the higher N concentrations in females inoculated with AMF than in males grown with AMF under saline conditions were further verified in our study as the indicator of positive mycorrhizal effect on female plants, which is in agreement with *Chrysanthemum morifolium* as observed by Wang et al. (2018). However, previous studies suggest that mycorrhizal efficacies under saline conditions are mainly related to mycorrhiza-induced P enhancement in the host (Al-Karaki, 2000; Wu et al., 2010; Chandrasekaran et al., 2014). The P concentrations of males inoculated with AMF were higher than those of females grown with AMF (Figure 5), suggesting that there were sex-specific mycorrhizal strategies in nutrient absorption under saline conditions. The improvement of nutrient absorption with AM fungi colonization can be attributed to the extensive absorption surface of roots with hyphal networks of AMF in the soil (Smith and Read, 2008).

Salt stress may induce plants with ionic imbalances in plant cells, which usually arise from nutrient availability, competitive uptake, transport, or partitioning within the plants (Munns and Tester, 2008; Wu et al., 2010). In this study, a significant increase in Na^+^, but with a significant decrease in K^+^, was detected with an increase in salinity. Na^+^ and K^+^ compete for the binding sites of enzymes that are essential for various cellular functions (Evelin et al., 2009; Kapoor et al., 2019). Otherwise, our study showed that AMF inoculation conferred more benefits on K^+^, Mg^2+^, Fe^2+^, Zn^2+^, and Mn^2+^ in female plants inoculated with AMF than in male plants grown with AMF (Figure 5), which was consistent with previous studies, and will help plants to limit cellular Na^+^ accumulation to toxic levels (Giri et al., 2007; Wu et al., 2010). Notably, in this study, the effects of AMF on the maintenance of favorable K^+^/Na^+^, Ca^2+^/Na^+^, and Mg^2+^/Na^+^ ratios were evidently higher in the roots than in the shoots (Figure 6), which is in support of the view that AMF plays a more important role in the roots (Wu et al., 2016). Under saline conditions, root systems are the main plant organs that are subjected to salinity stress; therefore, higher K^+^/Na^+^, Ca^2+^/Na^+^, and Mg^2+^/Na^+^ ratios in roots with mycorrhizal inoculation would not fail to improve the salt tolerance of plants (Chandrasekaran et al., 2014). The abovementioned findings indicated that there were sex-specific differences in nutrient uptake. Nevertheless, female plants inoculated with AMF exhibited significantly higher Na^+^ concentrations in the shoots than male plants with mycorrhizal inoculation did under saline conditions, which are in agreement with the findings in *P. cathayana* (Chen et al., 2010). It is likely that the female plants possess an efficient mechanism for compartmentation of Na^+^ and/or partitioning of Na^+^ to old leaves that could not incur toxicity to plant growth.

Salinity affects not only the host plant but also the AMF. In the current experiment, AMF colonization peaked at 50 mM NaCl in both sexes and then decreased at 200 mM NaCl (Figure 1), indicating a dose dependence of salinity in modulating mycorrhizal colonization. This is in agreement with previous reports stating that AMF colonization does not decrease in the presence of NaCl (Aliasgharzad et al., 2001; Yamato et al., 2008). Evelin et al. (2009) suggested that whether or not the root colonization by AMF is reduced in the presence of NaCl is dependent on the timing of the observation, and there is more inhibition in the early than in the later stages of the symbiosis. Hence, it is probably that preinoculation of plants with AMF would help the host bypass the following inhibitory effects of mild salinity on spore germination. Moreover, the root AMF colonization of female plants was higher than that of male plants at lower saline conditions, which is consistent with the results of studies on *A. dioica* (Vega-Frutis et al., 2013) and *D. spicata* (Reuss-Schmidt et al., 2015) but contradictory to the results on *Populus deltoides* (Chen et al., 2016). Overall, our findings support the idea that root mycorrhizal colonization is saline dose dependent and sex specific.

Until now, some of the well-known mechanisms underlying the salt tolerance of mycorrhizal host plants involved the promotion of nutrient uptake, photosynthetic efficiency, WUE, osmoprotectant efficiency, antioxidant system, or maintenance of ionic homeostasis in host plants (Evelin et al., 2009; Chandrasekaran et al., 2014). These mechanisms always act in tandem to enhance the mycorrhizal host plants’ resistance to salinity stress (Kapoor et al., 2019). The results of the current study showed that mycorrhizal efficiency was positively correlated with Mn^2+^, proline, and N concentrations, suggesting that the mediation of these substances probably underlies the main mechanism that leads to enhanced salt tolerance in mycorrhizal plants. Moreover, we observed that females inoculated with AMF had higher nutrient concentrations and accumulated more biomass and osmolytes under saline conditions than males grown with AMF. Conversely, males did not accumulate more biomass but just distributed more biomass to their roots and increased some of the ionic ratios. Therefore, female plants with mycorrhizal inoculation adapted to saline conditions in an extravagant manner, whereas males inoculated with AMF adapted in a conservative manner, which is consistent with the findings of a greater mycorrhizal benefit observed in females in the dioecious *Carica papaya* (Vega-Frutis and Guevara, 2009) but countered what has been suggested in *Paulownia tomentosa* that both sexes equally benefit from the symbiotic relationship under low-salinity conditions (Lu et al., 2014). It is reported that the response of the host plant to AMF is dynamic in terms of costs and benefits and varies with plant species (Johnson et al., 1997). Hence, more investigations are needed to confirm the mechanisms underlying the sex-specific mycorrhizal strategies in dioecious *M. alba* under saline environments.

In conclusion, salt stress significantly limited the biomass production and nutrient concentrations of both *M. alba* sexes. Our results showed that AMF inoculation could alleviate the detrimental effects of salinity. Females benefited more from building a symbiotic relationship with *F. mosseae via* improving their biomass accumulation and N, proline, K^+^, Mg^2+^, Fe^2+^, Zn^2+^, and Mn^2+^ concentrations than males with mycorrhizal inoculation under saline conditions. Males responded to the mycorrhizal inoculation *via* higher root/shoot ratios and P and POD concentrations as well as lower shoot/root Na^+^ ratios than females inoculated with AMF under saline conditions. These sex-specific differences suggest that the two *M. alba* sexes adopted different mycorrhizal strategies when growing under saline conditions. Our findings highlight that female host plants respond in an extravagant manner, whereas male AM plants respond in a conservative manner under saline conditions. Besides *F. mosseae*, *Glomus fasciculatum* is also recorded to form mycorrhizal symbiosis with mulberry (Katiyar et al., 1995). It will be interesting to explore whether there are similar results as ours with other AM fungi because our research is conducted with only one AM fungus species. There is a large variety of AM fungi in nature; thus, further investigations involving different AM fungi on *M. alba* are deemed to be necessary for verifying our conclusions. Given the greater salt tolerance and positive mycorrhizal effects on female plants inoculated with *F. mosseae*, this biological approach can be recommended as a reliable and practical method for optimizing the yield in saline environments.

## Data Availability Statement

The raw data supporting the conclusions of this article will be made available by the authors, without undue reservation.

## Author Contributions

Y-HW designed the study, analyzed the data, wrote the first draft, and prepared the manuscript. N-LZ analyzed the data and prepared the manuscript. M-QW harvested samples and collected the data. X-BH collected the data and prepared the figures. Z-QL and JW provided the plant material. XS collected the data. YL and A-PW proposed the research, designed the study, and prepared the manuscript. All authors contributed to the article and approved the submitted version.

## Conflict of Interest

The authors declare that the research was conducted in the absence of any commercial or financial relationships that could be construed as a potential conflict of interest.

## Supplementary Material

Supplementary Figure 1 Colonization of arbuscular mycorrhizal fungus in the roots of male plants stained with acid fuchsin **(A)** and female plants stained with Trypan blue **(B)** of *Morus alba* under saline conditions.

Supplementary Figure 2 Effects of arbuscular mycorrhizal fungus on mycorrhizal growth response (MGR) **(A)** and salinity tolerance (ST) **(B)** in males and females of *Morus alba* under saline conditions. NM and AM represent inoculation with no mycorrhizal fungi and with *Funneliformis mosseae*, respectively. Values are presented as means ± SD (*n* = 6). Two-way ANOVA is performed to compare the effects of sex, salt, and their interactions on MGR in both males and females. Meanwhile, three-way ANOVA is performed to compare the effects of sex, salt, and AM inoculation and their interactions on ST in both males and females. Different letters indicate a significant difference according to least significant difference at *p* < 0.05.

Supplementary Figure 3 Principal component analysis of growth, photosynthetic properties, biochemical parameters, nutrient concentrations, and the treatment groups of salinity, sex, and arbuscular mycorrhizal fungus. MC, mycorrhizal colonization; MGR, mycorrhizal growth response; *A*, net photosynthetic rate; *g*_*s*_, stomatal conductance; *E*, transpiration rate; WUE, instantaneous water use efficiency; TDW, total dry weight; RSR, root/shoot ratio; POD, peroxidase; Pro, proline; SP, soluble protein; SKNa, K^+^/Na^+^ ratio in shoot; RKNa, K^+^/Na^+^ ratio in root; SCaNa, Ca^2+^/Na^+^ ratio in shoot; RCaNa, Ca^2+^/Na^+^ ratio in root; SMgNa, Mg^2+^/Na^+^ ratio in shoot; RMgNa, Mg^2+^/Na^+^ ratio in root; NaR, shoot/root ratio of Na^+^. The codes for treatment groups are as follows: MCN, male plants under 0 mM NaCl and without mycorrhizal inoculation; MLN, male plants under 50 mM NaCl and without mycorrhizal inoculation; MHN, male plants under 200 mM NaCl and without mycorrhizal inoculation; MCA, male plants under 0 mM NaCl and with mycorrhizal inoculation; MLA, male plants under 50 mM NaCl and with mycorrhizal inoculation; MHA, male plants under 200 mM NaCl and with mycorrhizal inoculation; FCN, female plants under 0 mM NaCl and without mycorrhizal inoculation; FLN, female plants under 50 mM NaCl and without mycorrhizal inoculation; FHN, female plants under 200 mM NaCl and without mycorrhizal inoculation; FCA, female plants under 0 mM NaCl and with mycorrhizal inoculation; FLA, female plants under 50 mM NaCl and with mycorrhizal inoculation; FHA, female plants under 200 mM NaCl and with mycorrhizal inoculation.

Supplementary Table 1 *F* values of three-way ANOVA for the effects of sex, salt stress, and arbuscular mycorrhizal fungus and their interactive effects on the parameters of *Morus alba*.

Supplementary Table 2 *F* values of three-way ANOVA for the effects of sex, salt stress, and arbuscular mycorrhizal fungus and their interactive effects on the nutrient uptake of *Morus alba*.

## Abbreviations

*A*: leaf net photosynthetic rate
AM: inoculated with AMF
AMF: arbuscular mycorrhizal fungi
*E*: transpiration rate
*g_s_*: stomatal conductance
MGR: mycorrhizal growth response
NM plants: non-mycorrhizal plants
POD: peroxidase
SP: soluble protein
ST: salinity tolerance
WUE: instantaneous water use efficiency.
