# Supplementary figures and images for: Sex-Specific Differences in the Physiological and Biochemical Performance of Arbuscular Mycorrhizal Fungi-Inoculated Mulberry Clones Under Salinity Stress
Source: Front Plant Sci. 2021 Mar 18;12:614162. doi: 10.3389/fpls.2021.614162 (PMC8012686; doi:10.3389/fpls.2021.614162)

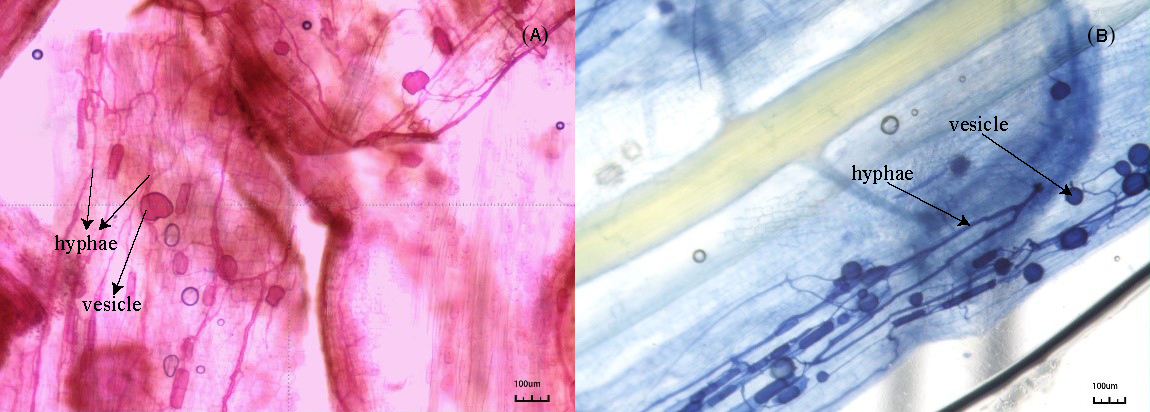

Supplement: Supplementary Figure 1 — Colonization of arbuscular mycorrhizal fungus in the roots of male plants stained with acid fuchsin (A) and female plants stained with Trypan blue (B) of Morus alba under saline conditions. [file Image_1.tif]

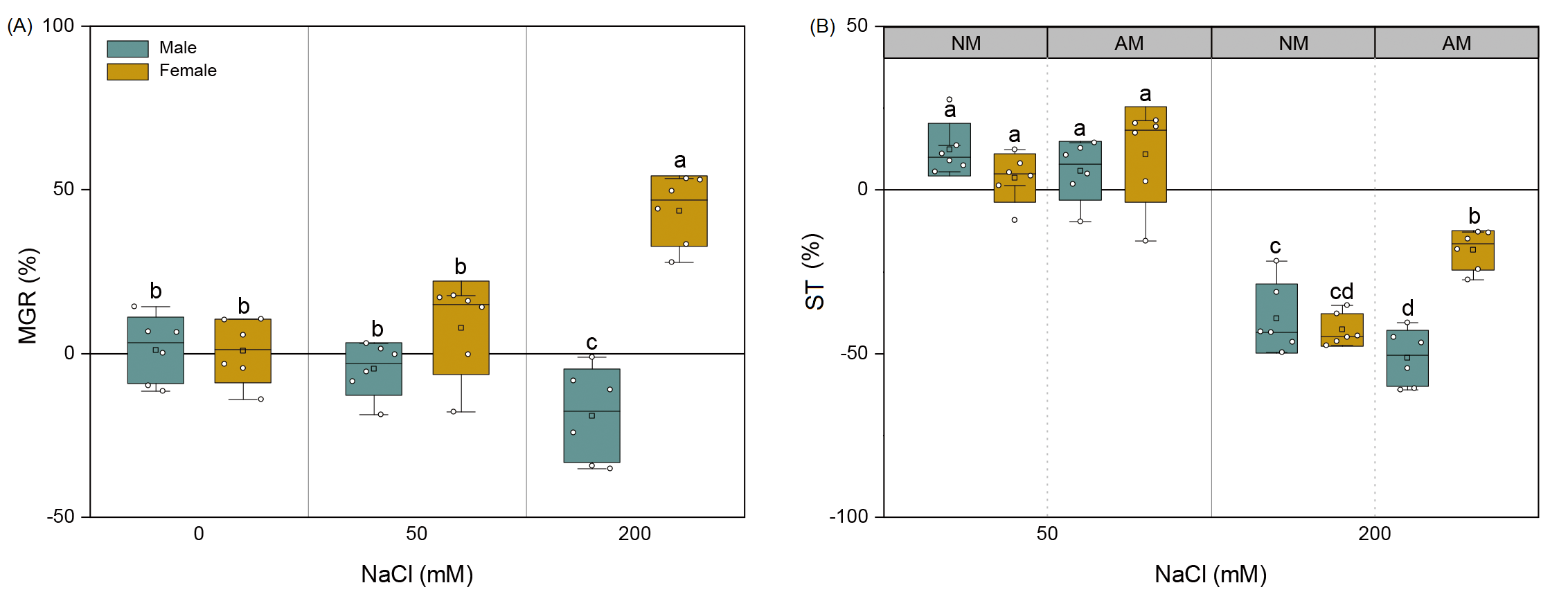

Supplement: Supplementary Figure 2 — Effects of arbuscular mycorrhizal fungus on mycorrhizal growth response (MGR) (A) and salinity tolerance (ST) (B) in males and females of Morus alba under saline conditions. NM and AM represent inoculation with no mycorrhizal fungi and with Funneliformis mosseae, respectively. Values are presented as means ± SD (n = 6). Two-way ANOVA is performed to compare the effects of sex, salt, and their interactions on MGR in both males and females. Meanwhile, three-way ANOVA is performed to compare the effects of sex, salt, and AM inoculation and their interactions on ST in both males and females. Different letters indicate a significant difference according to least significant difference at p < 0.05. [file Image_2.tif]

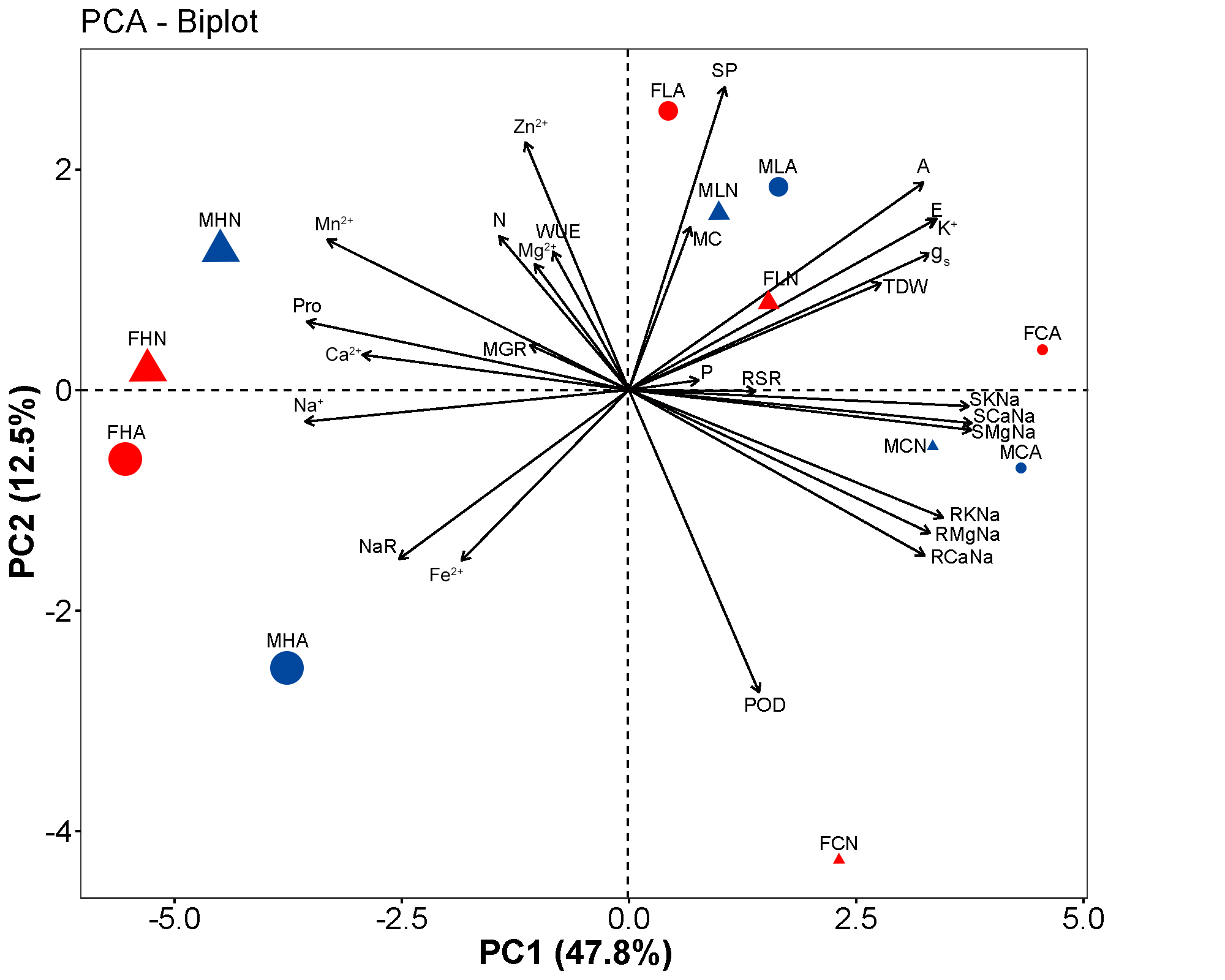

Supplement: Supplementary Figure 3 — Principal component analysis of growth, photosynthetic properties, biochemical parameters, nutrient concentrations, and the treatment groups of salinity, sex, and arbuscular mycorrhizal fungus. MC, mycorrhizal colonization; MGR, mycorrhizal growth response; A, net photosynthetic rate; gs, stomatal conductance; E, transpiration rate; WUE, instantaneous water use efficiency; TDW, total dry weight; RSR, root/shoot ratio; POD, peroxidase; Pro, proline; SP, soluble protein; SKNa, K+/Na+ ratio in shoot; RKNa, K+/Na+ ratio in root; SCaNa, Ca2+/Na+ ratio in shoot; RCaNa, Ca2+/Na+ ratio in root; SMgNa, Mg2+/Na+ ratio in shoot; RMgNa, Mg2+/Na+ ratio in root; NaR, shoot/root ratio of Na+. The codes for treatment groups are as follows: MCN, male plants under 0 mM NaCl and without mycorrhizal inoculation; MLN, male plants under 50 mM NaCl and without mycorrhizal inoculation; MHN, male plants under 200 mM NaCl and without mycorrhizal inoculation; MCA, male plants under 0 mM NaCl and with mycorrhizal inoculation; MLA, male plants under 50 mM NaCl and with mycorrhizal inoculation; MHA, male plants under 200 mM NaCl and with mycorrhizal inoculation; FCN, female plants under 0 mM NaCl and without mycorrhizal inoculation; FLN, female plants under 50 mM NaCl and without mycorrhizal inoculation; FHN, female plants under 200 mM NaCl and without mycorrhizal inoculation; FCA, female plants under 0 mM NaCl and with mycorrhizal inoculation; FLA, female plants under 50 mM NaCl and with mycorrhizal inoculation; FHA, female plants under 200 mM NaCl and with mycorrhizal inoculation. [file Image_3.tif]
