# Supplementary material for: Sex-Specific Differences in the Physiological and Biochemical Performance of Arbuscular Mycorrhizal Fungi-Inoculated Mulberry Clones Under Salinity Stress
Source: Front Plant Sci. 2021 Mar 18;12:614162. doi: 10.3389/fpls.2021.614162 (PMC8012686; doi:10.3389/fpls.2021.614162)
Supplement: Supplementary Table 1 — F values of three-way ANOVA for the effects of sex, salt stress, and arbuscular mycorrhizal fungus and their interactive effects on the parameters of Morus alba. [file Table_1.docx]

TABLE S1

*F* values of Three-way ANOVA for the effects of sex, salt stress, AM fungus and their interactive effects on the parameters of *Morus alba*

| **Variables** | **Sex** | **Salt** | **AMF** | **Sex×Salt** | **Sex×AMF** | **Salt×AMF** | **Sex×Salt×AMF** |
| --- | --- | --- | --- | --- | --- | --- | --- |
| Total dry weight (g) | 59.106^***^(1,60) | 150.358^***^(2,60) | 2.454^ns^(1,60) | 1.945^ns^(2,60) | 13.670^***^(1,60) | 0.957^ns^(2,60) | 5.713^**^(2,60) |
| Root:shoot ratio (g g^-1^)  *A* (µmol m^-2^ s^-1^) | 103.612^***^(1,60)  7.802^*^(1,24) | 9.487^***^(2,60)  145.613^***^(2,24) | 10.854^**^(1,60)  18.293^***^(1,24) | 2.077^ns^(2,60)  12.413^***^(2,24) | 1.478^ns^(1,60)  25.190^***^(1,24) | 4.571^*^(2,60)  15.110^***^(2,24) | 27.272^***^(2,60)  6.493^**^(2,24) |
| *E* (mol m^-2^ s^-1^) | 22.368^***^(1,24) | 271.868^***^(2,24) | 62.132^***^(1,24) | 10.103^***^(2,24) | 29.779^***^(1,24) | 23.838^***^(2,24) | 31.015^***^(2,24) |
| *g_s_* (mol m^-2^ s^-1^) | 8.405^**^(1,24) | 186.273^***^(2,24) | 58.300^***^(1,24) | 2.528^ns^(2,24) | 21.192^***^(1,24) | 25.956^***^(2,24) | 22.463^***^(2,24) |
| *WUE* | 3.132 ^ns^ (1,24) | 10.273^***^(2,24) | 0.253^ns^(1,24) | 1.508^ns^(2,24) | 5.353^*^(1,24) | 0.329^ns^(2,24) | 5.819^**^(2,24) |
| Proline (µg g^-1^ FW) | 362.360^***^(1,24) | 8130.806^***^(2,24) | 1067.618^***^(1,24) | 57.267^***^(2,24) | 121.176^***^(1,24) | 488.457^***^(2,24) | 144.961^***^(2,42) |
| SP (mg g^-1^ FW) | 146.723^***^(1,24) | 376.109^***^(2,24) | 0.738^ns^(1,24) | 12.592^***^(2,24) | 138.517^***^(1,24) | 20.825^***^(2,24) | 20.659^***^(2,24) |
| POD (U mg^-1^ protein) | 1210.332^***^(1,24) | 4346.702^***^(2,24) | 1745.672^***^(1,24) | 1968.633^***^(2,24) | 391.894^***^(1,24) | 6760.347^***^(2,24) | 1094.783^***^(2,24) |
| ST (%) | 6.074^*^(1,40) | 308.873^***^(1,40) | 1.526^ns^(1,40) | 9.968^**^(1,40) | 22.717^***^(1,40) | 1.209^ns^(1,40) | 4.651^*^(1,40) |

*F*-values are followed by *df* values in parentheses. *A*, *E*, *g_s_*, *WUE*, SP, POD and ST represent net photosynthetic rate, transpiration rate, stomatal conductance, water use efficiency, soluble protein, peroxidases and salt-tolerance index. Significance levels: ^*^ *p* $<$ 0.05; ^**^ *p* < 0.01; ^***^ *p* $\leq$ 0.001; *ns*, not significant at *p* > 0.05.
