# Supplementary material for: Sex-Specific Differences in the Physiological and Biochemical Performance of Arbuscular Mycorrhizal Fungi-Inoculated Mulberry Clones Under Salinity Stress
Source: Front Plant Sci. 2021 Mar 18;12:614162. doi: 10.3389/fpls.2021.614162 (PMC8012686; doi:10.3389/fpls.2021.614162)
Supplement: Supplementary Table 2 — F values of three-way ANOVA for the effects of sex, salt stress, and arbuscular mycorrhizal fungus and their interactive effects on the nutrient uptake of Morus alba. [file Table_2.docx]

TABLE S2

*F* values of Three-way ANOVA for the effects of sex, salt stress, AM fungus and their interactive effects on the nutrient uptake of *Morus alba*

| **Variables** | **Sex** | **Salt** | **AMF** | **Sex×Salt** | **Sex×AMF** | **Salt×AMF** | **Sex×Salt×AMF** |
| --- | --- | --- | --- | --- | --- | --- | --- |
| Sodium (Na^+^) (mg·g^-1^) | 321.034^***^(1,24) | 42369.266^***^(2,24) | 34.843^***^(1,24) | 221.370^***^(2,24) | 440.329^***^(1,24) | 72.341^***^(2,24) | 132.386^***^(2,42) |
| Nitrogen (N) (mg·g^-1^) | 188.166^***^(1,24) | 342.197^***^(2,24) | 21.293^***^(1,24) | 213.744^***^(2,24) | 154.821^***^(1,24) | 991.536^***^(2,24) | 85.670^***^(2,42) |
| Phosphorus (P) (mg·g^-1^) | 228.608^***^(1,24) | 2.001^ns^(1,24) | 21.293^***^(1,24) | 46.915^***^(2,24) | 274.621^***^(1,24) | 134.179^***^(2,24) | 38.752^***^(2,42) |
| Potassium (K^+^) (mg·g^-1^) | 0.610^ns^(1,24) | 2281.477^***^(2,24) | 53.209^***^(1,24) | 40.637^***^(2,24) | 192.121^***^(1,24) | 163.014^***^(2,24) | 44.654^***^(2,42) |
| Calcium (Ca^2+^) (mg·g^-1^) | 0.683^ns^(1,24) | 239.560^***^(2,24) | 13.369^***^(1,24) | 217.709^***^(2,24) | 5.215^*^(1,24) | 0.968^ns^(2,24) | 10.054^***^(2,42) |
| Magnesium (Mg^2+^) (mg·g^-1^) | 198.677^***^(1,24) | 24.667^***^(2,24) | 258.556^***^(1,24) | 101.484^***^(2,24) | 115.868^***^(1,24) | 66.271^***^(2,24) | 7.701^**^(2,42) |
| Iron (Fe^2+^) (mg·g^-1^) | 2922.237^***^(1,24) | 430.303^***^(2,24) | 862.683^***^(1,24) | 144.521^***^(2,24) | 1.105^ns^(1,24) | 792.654^***^(2,24) | 467.022^***^(2,42) |
| Zinc (Zn^2+^) (mg·g^-1^) | 76.736^***^(1,24) | 138.614^***^(2,24) | 1.046^ns^(1,24) | 4.959^*^(2,24) | 362.203^***^(1,24) | 754.948^***^(2,24) | 143.909^***^(2,42) |
| Manganese (Mn^2+^) (mg·g^-1^) | 210.389^***^(1,24) | 1200.149^***^(2,24) | 71.239^***^(1,24) | 129.140^***^(2,24) | 112.028^***^(1,24) | 133.869^***^(2,24) | 31.497^***^(2,42) |
| K^+^:Na^+^ ratio in shoot | 258.222^***^(1,24) | 21968.543^***^(2,24) | 9.235^***^(1,24) | 361.126^***^(2,24) | 24.494^***^(1,24) | 241.567^***^(2,24) | 34.134^***^(2,42) |
| K^+^:Na^+^ ratio in root | 1.192^ns^(1,24) | 15146.741^***^(2,24) | 238.715^***^(1,24) | 136.749^***^(2,24) | 214.082^***^(1,24) | 473.167^***^(2,24) | 116.507^***^(2,42) |
| Ca^2+^:Na^+^ ratio in shoot | 21.057^***^(1,24) | 11573.945^***^(2,24) | 199.770^***^(1,24) | 260.693^***^(2,24) | 131.320^***^(1,24) | 152.835^***^(2,24) | 94.295^***^(2,42) |
| Ca^2+^:Na^+^ ratio in root | 188.559^***^(1,24) | 10275.417^***^(2,24) | 359.731^***^(1,24) | 159.320^***^(2,24) | 190.236^***^(1,24) | 33.389^***^(2,24) | 98.672^***^(2,42) |
| Mg^2+^:Na^+^ ratio in shoot | 86.069^***^(1,24) | 11265.205^***^(2,24) | 171.444^***^(1,24) | 191.205^***^(2,24) | 81.401^***^(1,24) | 159.036^***^(2,24) | 41.367^***^(2,42) |
| Mg^2+^:Na^+^ ratio in root | 677.301^***^(1,24) | 11521.862^***^(2,24) | 6.651^*^(1,24) | 4.614^*^(2,24) | 2.937^ns^(1,24) | 11.746^***^(2,24) | 70.315^***^(2,42) |
| Shoot:root ratio of Na^+^ | 51.139^***^(1,24) | 720.882^***^(2,24) | 338.107^***^(1,24) | 19.238^***^(2,24) | 106.142^***^(1,24) | 255.568^***^(2,24) | 178.637^***^(2,42) |

*F*-values are followed by *df* values in parentheses. Significance levels: ^*^ *p* $<$ 0.05; ^**^ *p* < 0.01; ^***^ *p* $\leq$ 0.001; *ns*, not significant at *p* > 0.05.
